# Supplementary material for: Heat adaptation of phage T7 under an extended genetic code
Source: Virus Evol. 2021 Dec 1;7(2):veab100. doi: 10.1093/ve/veab100 (PMC8923235; doi:10.1093/ve/veab100)
Supplement: veab100_Supp [file veab100_supp.zip › Sequencing_Analysis.zip!/Sequencing_Analysis/A2/evidence/SNP_13.html]

BRESEQ :: Evidence


| Predicted mutation | | | | | | |
| --- | --- | --- | --- | --- | --- | --- |
| evidence | seq id | position | mutation | annotation | gene | description |
| RA | NC\_001604.1 | 34,975 | A→G | intergenic (–/–) | – / – | –/– |

| Read alignment evidence... | | | | | | | | | | | |
| --- | --- | --- | --- | --- | --- | --- | --- | --- | --- | --- | --- |
|  | seq id | position | | ref | new | freq | score (cons/poly) | reads | annotation | genes | product |
| \* | NC\_001604.1 | 34,975 | 0 | A | G | 98.9% | 1309.1 / NA | 434 | intergenic (–/–) | *–/–* | –/– |
| Reads supporting (aligned to +/- strand):  **ref** base A (0/1);  **major** base G (215/213);  **minor** base T (4/1);  **total** (219/215) | | | | | | | | | | | |
| Fisher's exact test for biased strand distribution *p*-value = 3.72e-01 | | | | | | | | | | | |
| Kolmogorov-Smirnov test that lower quality scores support variant *p*-value = 9.98e-01 | | | | | | | | | | | |

Only 100 of 434 total aligned reads displayed.  

`GATTTTAATATCCCGTTTGAGTATCTAGCCCGTAAGTTCGTAGTGGTAACTCTTATTGGTGTAGACCGAAAGGTCCTTACGATTAATACAGACTATCGCTTTGCTACACGTACTACTATCTCTCTGACAAAGGCTTGGGGTCCAGCCGATGGCTACACGACCATCGAGTTACGTCGAGTAACCTCCACTACCGACCGATTGGTTGACTTTACGGATGGTTCAATCCTCCGCGCGTATGACCTTAACGTCGCTCAGATTCAAACGATGCACGTAGCGGAAGAGGCCCGTGACCTCACTACGGATACTATCGGTGTCAATAACGATGGTCACTTGGATGCTCGTGGTCGTCGAATTGTGAACCTAGCGAACGCCGTGGATGACCGCGATGCTGTTCCGTTTGGTCAACTAAAGACCATGAACCAGAACTCATGGCAAGCACGTAATGAAGCCTTACAGTTCCGTAATGAGGCTGAGACTTTCAGAAACCAAGCGGAGGGCTTTAAGAACGAGTCCAGTACCAACGCTACGAACACAAAGCAGTGGCGCGATGAGACCAAGGGTTTCCGAGACGAAGCCAAGCGGTTCAAGAATACGG  >  NC_001604.1/34678‑35272`  

                                                                                                                                                                                                                                                                                                         `|`                                                                                                                                                                                                                                                                                                           
                           `cgtgggctcggagatgtgtataagagacagggTGTAGACCGAAAGGTCCTTACGATTAATACAGACTATCGCTTTGCTACACGTACTACTATCTCTCTGACAAAGGCTTGGGGTCCAGCCGATGGCTACACGACCATCTAGTTACGTCGAGTAACCTCCACTACCGACCGATTGGTTGACTTTACGGATGGTTCAATCCTCCGCGCGTATGACCTTAACGTCGCTCAGATTCAAACGATGCACGTAGCGGAAGAGGCCCGTGACCTCACTGCGGATACTATCGGTGTCAATAACGATGGTc                                                                                                                                                                                                                                                                             <  1:368127/271‑1 (MQ=255)`  
                             `ccGTAAGTTCGTAGTGGTAACTCTTATTGGTGTAGACCGAAAGGTCCTTACGATCAATACAGACTATCGCTTTGCTACACGTACTACTATCTCTCTGACAAAGGCTTGGGGTCCAGCCGAGGGCTACACAACCATCGAGTTACGTCGAGTAACCTCCACTACCGACCGATTGGTTGACTTTACGGATGGTTCAATCCTCCGCGCGTATGACCTTAACGTCGCTCAGATTCAAACGATGCACGTAGCGGAAGAGGCCCGTGACCTCACTGCGGATACTATCGGTGTCAATAACGATGGTCAc                                                                                                                                                                                                                                                                           <  1:641682/301‑1 (MQ=255)`  
                              `cGTAAGTTCGTAGTGGTAACTCTTATTGGTGTAGACCGAAAGGTCCTTACGATTAATACAGACTATCGCTTTGCTACACGTACTACTATCTCTCTGACAAATGCTTGGGGTCCAGCCGATGGCTACACGACCATCGAGTTACGTCGAGTAACCTCCACTACCGACCGATTGGTTGACTTTACGGATGGTTCAATCCTCCGCGCGTATGACCTTAACGTCGCTCAGATTCAAACGATGCACGTAGCGGAAGAGGCCCGTGACCTAACTGCGGATACTATCGGTGTCAATAACGATGGTCACt                                                                                                                                                                                                                                                                          >  1:186125/1‑301 (MQ=255)`  
                                    `ttCGTAGTGGTAACTCTTATTGGGGTAGACCGAAAGGTCCTTACGATTAATACAGACTATCGCTTTGCTACACGTACTACTATCTCTCTGACAAAGGCTTGGGGTCCAGCCGATGGCTACACGACCATCGAGTTACGTCGAGTAACCTCCACTACCGACCGATTGGTTGACTTTACGGATGGTTCAATCCTCCGTGCGTATGACCTTAACGTCGCTCAGATTCAAACGATGCACGTAGCGGAAGAGGCCCGTGACCTCACTGCGGATACTATCGGTGTCAATAACGATGGTCACTTGGATg                                                                                                                                                                                                                                                                    <  1:124526/301‑1 (MQ=255)`  
                                     `tCGTAGTGGTAACTCTTATTGGTGTAGACCGAAAGGTCCTTACGATTAATACAGACTATCGCTTTGCTACACGTACTACTATCTCTCTGACAAAGGCTTGGGGTACAGCCGATGGCTACACGACCATCGAGTTACGTCGAGTAACCTCCACTACCGACCGATTGGTTGACTTTACGGATGGTTCAATCCTCCGCGCGTATGACCTTAACGTCGCTCAGATTCAAACGATGCACGTAGCGGAAGAGGCACGTGACCTCACTGCGGATACTATCAGTGTCAATAACGATGGAAACTTGGATGc                                                                                                                                                                                                                                                                   <  1:71156/301‑1 (MQ=255)`  
                                          `gTGGTAACTCTTATTGGTGTAGACCGAAAGGTCCTTACGATTAATACAGACTATCGCTTTGCTACACGTACTACTATCTCTCTGACAAAGGCTTGGGGTCCAGCCGATGGCTACACGACCATCGAGTTACGTCGAGTAACCTCCACTACCGACCGATTGGTTGACTTTACGGATGGTTCAATCCTCCGCTCGTATGACCTTAACGTCGCTCAGATTCAAACGATGCAAGTAGTGGAAGAGTCCCGTGACCTCACTGCGGATACTATCGGTGTCAATAACGATGGTCACTCGGATGCTCCTg                                                                                                                                                                                                                                                              >  1:157708/1‑301 (MQ=255)`  
                                           `tGGTAACTCTTATTGGTGTAGACCGAAAGGTCCTTACGATTAATACAGACTATCGCTTTGCTACACGTACTACTATCTCTCTGACAAAGGCTTGGGGTCCAGCCGATGGCTACACGACCATCGAGTTACGTCGAGTAACCTCCACTACCGACCGATTGGTTCACTTTACTGATGGTTCAATCCTCCGCGCGTATGACCTTAACGTCGCTCAGATTCAAACGATGCACGTAGCGGAAGAGGCCCGTGACCTTACTGCTGATACTATCGGTTTCAATAACGATGGTCACTTGGATGCTCGTgg                                                                                                                                                                                                                                                             >  1:135788/1‑301 (MQ=255)`  
                                             `gTAACTCTTATTGGTGTAGACCGAAAGGTCCTTACGATTAATACAGACTATCGCTTTGCTACACGTACTACTATCTCTCTGACAAAGGCTTGGGGTCCAGCCGATGGCTACACGACCATCGAGTTACGTCGAGTAACCTCCACTACCGACCGATTGGTTGACTTTACGGATGGTTCAATCCTCCGCGCGTATGACCTTAACGTCGCTCAGATTCAAACGATGCACGTAGCGGAAGAGTCCCGTGACCTCACTGCGGATATTATCGGTGTCAATAACGATGGTCACTTGGAtctgtctcttt                                                                                                                                                                                                                                                           >  1:101251/1‑291 (MQ=255)`  
                                             `gTAACTCTTATTGGTGTAGACCGAAAGGTCCTTACGATTAATACAGACTATCGCTTTGCTACACCTACTACTATCGCTCTGACAAAGGCTTGGGGTCCAGCCGATGGCTACACGACAATCGAGTTACGTCGAGTAACCTCCACTACCGACCCGTTAATTGCCTTTACGGATGGTTCAGTCCTCCGCGCGTATGCCCTTAACGTCGCTCAGATTCAAACGATGCACGTAGCGGAAGAGGCCAGTGACCTCACTGCGGATACTATCGGTGTCAATAACGATGGTCACTTGGATGCTCGTGgtc                                                                                                                                                                                                                                                           <  1:39965/301‑1 (MQ=255)`  
                                                 `ctctTATTGGTGTAGACCGAAAGGTCCTTACGATTAATACAGACTATCGCTTTGCTACACGTACTACTATCTCTCTGACAAAGGCTTGGGTTCCAGCCGATGGCTACACGACCATCGAGTTACGTCGAGTAACCTCCACTACCGACCGATTGGTTGACTTTACGGATGGTTCAATCCTCCGCGCGTATGACCTTAACGTCGCTCAGATTCAAACGATGCACGTAGCGGAAGAGGCCCGTGACCTCACTGCGGATACTATCGGTGTCAATAACGATGGTCACTTGGATGCTCGTGGtcgtcg                                                                                                                                                                                                                                                       >  1:730863/1‑301 (MQ=255)`  
                                                 `ctctTATTGGTGTAGACCGAAAGGTCCTTACGATTAATACAGACTATCGCTTTGCTACACGTACTACTATCTCTCTGACAAAGGCTTGGGGTCCAGCCGATGGCTACACGACCATCGAGTTACGTCGAGTAACCTCCACTACCGACCGATTGGTTGACTTTACGGATGGTTCAATCCTCCGCGCGTATGACCTTAACGTCGCTCAGATTCAAACGATGCACGTAGCGGAAGAGGCCCGTGACCTCACTGCGGATACTATCGGTGTCAATAACGATGGTCACTTGGAtctgtctcttataca                                                                                                                                                                                                                                                       >  1:509376/1‑287 (MQ=255)`  
                                                 `ctctTATTGGTGTAGACCGAAAGGTCCTTACGATTAATACAGACTATCGCTTTGCTACACGTACTACTATCTCTATGACAAAGGCTTGGGGTCCAGCCGATGTCTACACGACCATCGAGCTACGTCGAGTAACCTCCACTACCGACCGATTGGTTGACTTTACGGATGGTTCAATCCTCAGCGCGTATGACATTAACGTCCCTCAGATTCACACGATGCACGTAGCGGAAGAGGCCCGTGACCTCACTGCGGATACTATCGGTGTCAATAACGATGGTCACTTGGATGCTCGTGGtcgtcg                                                                                                                                                                                                                                                       >  1:652805/1‑301 (MQ=255)`  
                                                         `ggTGTAGACCGAAAGGTCCTTACGATTAATACAGACTATCGCTTTGCTACACGTACTACTATCTCTCTGACAAAGGCTTGGGGTCCAGCCGATGGCTACACGACCATCGAGTTACGTCGAGTAACCTCCACTACCGACCGATTGGTTGACTTTACGGATGGTTCAATCCTCCGCGCGTATGACCTTAACGTCGCTCAGATTCAAACGATGCACGTAGCGGAAGAGGCCCGTGACCTCACTGCGGATACTATCGGTGTCAATAACGACTGTCTCttatacacatctccgagcccacgcgacc                                                                                                                                                                                                                                               >  1:787043/1‑275 (MQ=255)`  
                                                         `ggTGTAGACCCAAAGGTCCTTACGATTAATACAGACTATCGCTTTGCTACACGTACTACTATCCCTCTGACAAAGGCTTGGGGTCCAGCCGATGGCTACACGACCATCGAGTTACGTCGAGTAACCTCCACTACCGACCGATTGGTTGACTTTACGGATGGTTCAATCCTCCGCGCGTATGACCTTAACGTCGCTAAGATTCAAAAGATGCACGTAGCGGAAAAGGCCCGTGCACTCACTGCGGATACAATCGGTGTCAATAACAAGGGTCACTTGGATGATCGTGGTCGTCGAATtgtaa                                                                                                                                                                                                                                               >  1:620644/1‑299 (MQ=255)`  
                                                          `gtgtAGACCGAAAGGTCCTTACGATTAATACAGAATATCGCTTTGCTACACGTACTACTATCTCGCTGACAAAGGCTTGGGGTCCAGCCGATGGCTAAACGACCATCGAGTTACGTCGAGTAACCTCAACTACCGACCGATTGGTTGACTTTACGGATGGTTCAATCCTCCGCGCGTATGACCTTAACGTAGCTCAGATTCAAACGATGCACGTAGCGGAAGAGGCCCGTGACATCACTGCGGATACTATCGGTGTCAATAACGATGGTCACTTGGATGCTCGTGGTCGTCGAATTGTGaa                                                                                                                                                                                                                                              >  1:203716/1‑301 (MQ=255)`  
                                                           `tgtAGTCCGAAATGTCCTTACGATTAATACAGACTATCGCTTTGCTACACGTACTACTATCTCTCTGACAAAGGCTTGGGGTCCAGCCGATGGCTACACGACCATCGAGTTACGTCGAGTAACCTCCACTACCGACCGATTGGTTGACTTTACGGATGGTTCAATCCTCCGCGCGTATGACCTTAACGTCGCTCAGATTCAAACGATGCACGTAGCGGAAGAGGCCCGTGACCTCACTGCGGATACTATCGGTGTCAATAACGATGGTCACTTGGATGCTCGTGGTCGTCGAATTGTGAAc                                                                                                                                                                                                                                             <  1:128516/301‑1 (MQ=255)`  
                                                             `agatgtgtataagagacaggATTAATACAGACTATTGCTTTGCTACACGTACTACTATCTCTCTGACAAAGGCTTGGGGTCCAGCCGATGGCTACACGACCATCGAGTTACGTCGAGTAACCTCCACTACCGACCGATTGGTTGACTTTACGGATGGTTCAATCCTCCGCGCGTATGACCTTAACGTCGCTCAGATTCAAACGATGCACGTAGCGGAAGAGGCCCGTGACCTCACTGCGGATACTATCGGTGTCAATAACGATGGTCACTTGGATGCTCGTGGTCGTCGAATTGTGAACCt                                                                                                                                                                                                                                           <  1:695934/282‑1 (MQ=255)`  
                                                                `aCCGAAAGGTCCTTACGATTAATACAGACTATCGCTTTGCTACACGTACTACTATCTCTCTGACAAAGGCTTGGGGTCCAGCCGATGGCTACACGACCATCGAGTTACGTCGAGTAACCTCCACTACCGACCGATTGGTTGACTTTACGGATGGTTCAATCCTCCGCGCGTATGACCTTAACGTCGCTCAGATTCAAACGATGCACGTAGCGGAAGAGGCCCGTGACCTCACTGCGGATACTATCGGTGTCAATAACGATGGTACCTTGGAtctgtctcttatacacatctccgcgcccac                                                                                                                                                                                                                                        >  1:447770/1‑272 (MQ=255)`  
                                                                      `aGGTCCTTACGATTAATACCGACTTTCGCTTTGCTACACGTACTACTATCTCTCTGACAAAGGCTTGGGGTCCAGCCGATGGCTACACGACCATCGAGTTACGTCGAGTAACCTCCACTACCGACCAATTGGTTGACTTTACGGATGGTTCAATTCTCCGCGCGTATGACCTTAACGTCGCTCAGATTCAAACGATGCACGTAGCGGAAGAGGCCCGTGACCTCACTGCGGATACTATCGGTGTCAATAACGATGGTCACTTGGATGCTCGTGGTCGTCGAATTGTGAACCTAGCGAACGc                                                                                                                                                                                                                                  <  1:671908/301‑1 (MQ=255)`  
                                                                      `aGGTCCTTACGATTAATACAGACTATCGCTTTGCTACACGTACTACTATCTCTCTGACAAAGGCTTGGGTTCCAGCCGATGGCTACACGACCATCGAGTTACGTCGAGTAACCTCCACTACCGACCGATTGGTTGACTTTACTGATGGTTCAATCCTCCGCTCGTATGACCTTAACGTCGCTCAGATTCAAACGATGCACGTAGCGGAAGAGGCCCGTGACCTCACTGCGGATACTATCGGTGTCAATAACGATGGTCACTTGGATGCTCGTGGTCGTCGAATTGTGAACCTAGCAAACGc                                                                                                                                                                                                                                  <  1:255136/301‑1 (MQ=255)`  
                                                                       `ggTCCTTACGATTAATACAGACTATCGCTTTGCTACACGTACTACTATCTCTCTGACAAAGGCTTGGGGTCCAGCCGATGGCTACACGACCATCGAGTTACGTCGAGTAACCTCCACTACCGACCGATTGGTTGACTTTACTGATGGTTCAATCCTCCGCGCGTATGACCTTAACGTCGCTCAGGTTCAAACGATGCACGTAGCGGAAGAGGCCCGTGACCTCACTGCGGATACTATCGGTGTCAATAACGATGGTCACTTGGATGCTCGTGGTCGTCGAATTGTGAACCTAGCGAACGcc                                                                                                                                                                                                                                 >  1:705300/1‑301 (MQ=255)`  
                                                                        `gTCCTTACGATTAATACAGACTATCGCTTTGCTACACGTACTACTATCTCTCTGACAAAGGCTTGGGGTCCAGCCGATGGCTACACGACCATCGAGTTACGTCGAGTAACCTCCACTACCGACCGATTGGTTGACTTTACGGATGGTTCAATCCTCCGCGCGTATGACCTTAACGTCGCTCAGATTCAAACGATGCACGTAGCGGAAGAGGCCCGTGACCTCACTGCGGATACTATCGGTGTCAATAACGATGGTCACTTGGATGCTCATGgtcctgtctcttatacacatctccgggccc                                                                                                                                                                                                                                >  1:407894/1‑274 (MQ=255)`  
                                                                            `ttACGATTAATACAGACTATCGCTTTGCTACACGTACTACTATCTCTCTGACAAAGGCTTGGGGTCCAGCCGATGGCTACACGACCATCGAGTTACGTCGAGTAACCTCCACTACCGACCGATTGGTTGACTTTACGGATGGTTCAATCCTCCGCGCGTATGACCTTAACGTCGCTCAGATTCAAACGATGCACGTAGCGGAAGAGGCCCGTGACCTCACTGCGGATACTATCGGTGTCAATAACGATGGTCACTTGGATGCTCGTGGTCGTCGAATTGTGAACCTAGCGAACGCAGTGGa                                                                                                                                                                                                                            <  1:490028/301‑1 (MQ=255)`  
                                                                                `gATTAATACAGACTATCGCTTTGCTACACGTACTACTATCTCTCTGACAAATGCTTGGGGTCAAGCCGATGTCTACACGACCATCGAGTTACGTCGAGTAACCTCCACTACCGACCGATTGGTTGACTTTACGGATGGTTCAATCCTCCGCGCGTATGACCTTAACGTCGCTCAGATTCAAACGATGCACGTAGCGGAAGAGGCCCGTGACCTAACTGCGGATACTATCTGTGTCAATAACGATGGTCACTTGGATGCTCATGGTCGTCGCATTGTGACCCGAGCGAACGACGTGGATGAc                                                                                                                                                                                                                        >  1:770740/1‑301 (MQ=255)`  
                                                                                   `ggggctcggagatgtgtataagagacagactactATCTCTCTGACAAAGGCTTGGGGTCCAGCCGATGGCTACACGACCATCGAGTTACGTCGAGTAACCTCCACTACCGACCGATTGGTTGACTTTACGGATGGTTCAATCCTCCGCGCGTATGACCTTAACGTCGCTCAGATTCAAACGATGCACGTAGCGGAAGAGGCCCGTGACCTCACTGCGGATACTATCGGTGTCAATAACGATGGTCACTTGGATGCTCGTGGTCGTCGAATTGTGAACCTAGCGAACGCCGTGGATGACcgc                                                                                                                                                                                                                     <  1:441034/273‑1 (MQ=255)`  
                                                                                    `aaTACAGACTATCGCTTTGCTACACGTACTACTATCTCTCTGACAAAGGCTTGGGGTCCAGCCGATGGCTACACGACCATCGAGTTACGTCGAGTAACCTCCACTACCGACCGATTGGTTGACTTTACGGATGGTTCAATCCTCCGCGCGTATGACCTTAACGTCGCTCAGATTCAAACTATGCACGTAGCGGAAGATGCCCGTGACCTCACTGCGGATACTATCGGTGTCAATAACGATGGTCACTTGGATGCTCGTGGTCGTCGAATTGTGAACCtgtctcttatacacatctccgagc                                                                                                                                                                                                                    >  1:782546/1‑278 (MQ=255)`  
                                                                                     `aTACAGACTATCGCTTTGCTACACGTACTACTATCTCTCTGACAAAGGCTTGGGGTCCAGCCGATGGCTACACGACCATCGAGTTACGTCGAGTAACCTCCACTACCGACCGATTGGTTGACTTTACGGATGGTTCAATCCTCCGCGCGTATGACCTTAACGTCGCTCAGATTCAAACGATGCACGTAGCGGAAGAGGCCCGTGACCTCACTGCGGATACTATCGGTGTCAATAACGATGGTCACTTGGATGCTCGTGGTCGTCGAATTGTGAACCTAGCGAACGCCGTGGATGACCGCGa                                                                                                                                                                                                                   >  1:247813/1‑301 (MQ=255)`  
                                                                                        `cAGACTATCGCTTTGCTACACGTACTACTATCTCTCTGACAAAGGCTTGGGGTCCAGCCGATGGCTACACGACCATCGAGTTACGTCGAGTAACCTCCACTACCGACCGATTGGTTGACTTTACGGATGGTTCAATCCTCCGCGCGTATGACCTTAACGTCGCTCAGATTCAAACGATGCACGTAGCGGAAGAGGCACGTGACCTCACTGCGGATACTATCGGTGTCAATAACGATGGTCACTTGGATGCTCGTGGTCGTCGAATTGTGAACCTAGCGAACGCCGTGGATGACCGCGATGc                                                                                                                                                                                                                <  1:283636/301‑1 (MQ=255)`  
                                                                                        `cAGACTATCGCTTTGCTACACGTACTACTATCTCTCTGACAAAGGCTTGGGGTCCAGCCGATGGCTACACGACCATCGAGTTACGTCGAGTAACCTCCACTACCGACAGATTGGTTGACTTTACGGATGGTTCAATCCTCCGCGCGTATGACCTTAACGTCGCTAAGATTCAAACGATGCACGTAGCGGAAGAGGCCCGTGACCTCACTGCGGATACTATCGGTGTCAATAACGATGGTCACTTGGATGCTCGTGGTCGTCGAATTGTGAACCTAGCGAACGCCGTGGATGACCGCGATGc                                                                                                                                                                                                                <  1:193617/301‑1 (MQ=255)`  
                                                                                            `cTATCGCTTTGCTACACGTACTACTATCTCTCTGACAAAGGCTTGGGGTCCAGCCGATGGCTACACGACCATCGAGTTACGTCGAGTAACCTCCACTACCGACCGATTGGTTGACTTTACGGATGGTTCAATCCTCAGCGCGTATGACCTTAACGTCGCTCAGATTCAAACGATGCACGTAGCGGAAGAGGCCCGTGACCTCACTGCGGATACTATCGGTGTCAATAACGATGGTCACTTGGATGCTCGTGGTCGTCGAATTGTGAACCTAGCGAACGCCGTGGATGACCGCGATGCTGtt                                                                                                                                                                                                            >  1:219968/1‑301 (MQ=255)`  
                                                                                                      `gCTACACGTACTACTATCTCTCTGACAAAGGCTTGGGGTCCAGCCGATGGCTACACGACCATCGAGTTACGTCGAGTAACCTCCACTACCGACCGATTGGTTGACTTTACGGATGGTTAAATCCTCCGCGCGTATGACCTTAACGTCGCTCAGATTCAAACGATGCACGTAGCGGAAGAGGCACGTGACCTCACTGCGGATACTATCGGTGTCAATAACGATGGTCACTTGGATGCTAGTGGTCGTCGAATTGTGAAACTAGCGAACGCCGTGGATGACCGCGATGCAGTTCCGTTTGGTc                                                                                                                                                                                                  >  1:85518/1‑301 (MQ=255)`  
                                                                                                      `gCTACACGTACTACTATCTCTCTGACAAAGGCTTGGGGTCCAGCCGATGGCTACACGACCATCGAGTTACGTCGAGTAACCTACACTACCGACCGATTGGTTGACTTTACGGATGGTTCAATCCTCCGCGCGTATGACCTTAACGTCGCTCAGATTCAAACGATGCACGTAGCGGAAGAGGCCCGTGACCTCACTGCGGATACTATCGGTGTCAATAACGATGGTCACTTGGATGCTCGTGGTCGTCGAATTGTGAACCTAGCGAACGCCGTGGATGACCGCGATGCTGTTCCGTTTGGTc                                                                                                                                                                                                  >  1:664161/1‑301 (MQ=255)`  
                                                                                                      `gCTACACGTACTACTATCTCTCTGACAAAGGCTTGGGGTCCAGCCGATGGCTACACGAACATCGAGTTACGTCGAGTAACCTCCACTACCGACCGATTGGTTGACTTTAAGGATGGTTCAATCCTCCGCGCGTATGACCTTAACGTCGCTCAGATTCAAACGAAGCACGTAGCGGAAGAGGCCCGTGACCTCAATGCGGATACTATCGGTGTCAATAACGATGGTCACTTGGATGCTCGTGGTCGTCGAATTGTGAACCTAGCGAACGCCGTGGATGAcctgtcgcttataaacatctccg                                                                                                                                                                                                  >  1:420960/1‑280 (MQ=255)`  
                                                                                                         `acacGTACTACTATCTCTCTGACAAAGGCTTGGGGTCCAGCCGATGGCTACACGACCATCGAGTTACGTCGAGTAACCTCCACTACCGACTGATTGGTTGACTTTACGGATGATTCAATCCTCCGCGCGTATGACCTTAACGTCGCTCAGATTCAAACGATGCACGTAGCGGAAGAGGCCCGTGACCTCACTGCGGATACTATCGGTGTCAATAACGATGGTCACTTGGATGCTCGTGGTCGTCGAATTGTGAACCTAGCGAACGCCGTGGATGACCGCGATGCTGTTCCGTTTGGTCAAc                                                                                                                                                                                               >  1:249685/1‑301 (MQ=255)`  
                                                                                                           `acGTACTACTATCTCTCTGACAAAGGCTTGGGGTCCAGCCGATGGCTACACGACCATCGAGTTACGTCTAGTAACCTCCACTACCGACCGATTGGTTGACTTTACGGATGGTTCAATCCTCTGCGCGTATGACCTTAACGTCGCTCAGATTCAAACGATGCACGTAGCGGAAGAGGCCCGTGACCTTACTGCGGATACTATCTGTGTCAATAACGATGGTCACTTGGATGCTCGTGGTCGTCGAATTGTGAACCTAGCGAACGCCGTGGTTGACCGCGATGCTGTTCCGTTTGGTCAACTa                                                                                                                                                                                             >  1:241276/1‑301 (MQ=255)`  
                                                                                                            `cGTACTACTATCTCTCTGACAAAGGCTTGGGGTCCAGCCGATGGCTACACGACCATCGAGTTACGTCGAGTAACCTCCACTACCGACCGATTGGTTGACTTTACGGATGGTTCAATCCTCAGCGCGTATGACCTTAACGTCGCTCAGATTCAAACGATGCACGTAGCGGAAGAGGCCCGTGACCTCACTGCGGATACTATCGGTGTCAATAACGATGGTCACTTGGATGCTCGTGGTCGTCGAATTGTGAACCTAGCGAACGCCGTGGATGACCGCGATGCTGTTCCGTTTGGTAAACTaa                                                                                                                                                                                            <  1:200342/301‑1 (MQ=255)`  
                                                                                                              `tactacTATCTCTCTGACAAAGGCTTGGGGTACAGCCGATGGCTACACGACCATCGAGTTACGTCGAGTAACCTCCACTACCGACAGATTGGTTGACTTTACGGATGGTTCAATCCTCCGCGCGTATGACCTTAACGTCGCTCAGATTCAAACGATGCACGTAGCGGAAGAGGCCCGTGACCTCACTGCGGATACTATCGGTGTCAATAACGATGGTCACTTGGATGCTCGTGGTCGTCGAATTGTGAACCTAGCGAACGCCGTGGATGAACGCGGTGCTGTTCCGGTTGGTCAACTAAAg                                                                                                                                                                                          >  1:540540/1‑301 (MQ=255)`  
                                                                                                                `ctactaTCTCTCTGACAAAGGCTTGGGGTCCAGCCGATGGCTACACGACCATCGAGTTACGTCGAGTAACCTCCACTACCGACCGATTGGTTGACTTTACGGATGGTTCAATCCTCCGCGCGTATGACCTTAACGTCGCTCAGATTCAAACGATGCACGTAGCGGAAGAGGCCCGTGACCTCACTGCGGATACTATCGGTGTCAATAACGATGGTCACTTGGATGCTCGTGGTCGTCGAATTGTGAACCTAGCGAACGCCGTGGATGACCGCGATGCTGTTCCGTTTGGTCAACTAAAGAc                                                                                                                                                                                        <  1:230927/301‑1 (MQ=255)`  
                                                                                                                `ctactaTCTCTCTGACAAAGGCTTGGGGTCCAGCCGATGGCTACACGACCATCGAGTTACGTCGAGTAACCTCCACTACCGACCGATTGGTTGACTTTACGGATGGTTCAATCCTCCGCGCGTATGACCTTAACGTCGCTCAGATTCAAACGATGCACGTAGCGGAAGAGGCCCGTGACCTCACTGCGGATACTATCGGTATCAATAACGATGGTCACTTGGATGCTCGTAGTAGTCGAATTGTGAACCTAGCGAACGCCGTGGATGACCGCGATGCTGTTCCGTTTGGTCAACTAAAGAc                                                                                                                                                                                        >  1:570807/1‑301 (MQ=255)`  
                                                                                                                       `ctctctGACAAAGGCTTTGGGTCCAGCCGATGGCTACACGACCATCGAGTTACGTCGAGTAACCTCCACTACCGACCGATTGGTTGACTTTACTGATGGTTCAATCCTCCGCGCGTATGACCTTAACGTCGCTCAGATTCAAACGATGCACGTAGCGGAAGAGGCCCGTGACCTCACTGCGGATACTATCGGTGTCAATAACGATGGTCACTTGGATGCTCGTGGTCGTCGAATTGTGAACCTAGCGAACGCCGTGGATGACCGCGATGCTGTTCCGTTTGGTCAACTAAAGACCATGGAc                                                                                                                                                                                 >  1:60226/1‑301 (MQ=255)`  
                                                                                                                             `gACAAAGGCTTGGGGTCCATCCGATGGCTACACGACCATCGAGTTACGTCGAGTAACCTCCACTACCGACCGATTGGTTGACTTTACGGATGATTCAATCCTCCGCGCGTATGACCTTAACGTCGCTCAGATTCAAACGATGCACGTAGCGGAAGAGGCCCGTGACCTCACTGCGGATACTATCGGTGTCAATAACGATGGTCACTTGGATGCTCGTGGTCGTCGAATTGTGAACCTAGCGAACGCCGTGGATGACCGCGATGCTGTTCCGTTTGGTCAACTAAAGACCATGGACCAGAAc                                                                                                                                                                           >  1:562543/1‑301 (MQ=255)`  
                                                                                                                             `gACAAAGGCTTGGGGTCCAGCCGATTGCTACACGACCATCGAGTTACGTCGAGTAACCTCCACTACCGACCGATTGGTTGACTTTACGGATGGTTCAATCCTCCGCGCGTATGACCTTAACGTCGCTCAGATTCAAACGATGCACGTAGCGGAAGAGGCCCGTGACCTCACTGCGGATACTATCGGTGTCAATAACGATGGTCACTTGGATGCTCGTGGACGTCGAATTGTGAACCTAGCGAACGCCGGGGATGACCGCGATGCTGTTCCGGTTTGTCAACAAAAAACCAGCGACCAGaaa                                                                                                                                                                           >  1:583633/1‑300 (MQ=255)`  
                                                                                                                             `gACAAAGGCTTGGGGTCCAGCCGATGGCTACACGACCATCGAGTTACGTCGAGTAACCTCCACTACCGACCGATTGGTTTACTTTACGGATGGTTCAATCCTCCGCGCGTATGACCTTAACGTCGCTCAGATTCAAACGATGCACGTAGCTGAAGAGGCCCGTGACCTCACTGCGGATACTATCGGTGTCAATAACGATGGTCACTTGGATGCTCGTGGTCGTCGAATTGTGAACCTAGCGAACGCCGTGGATGACCGCGATGCTGTTCCGTTTGGTCAACTAAAGACCATGGACCAGAAc                                                                                                                                                                           <  1:273944/301‑1 (MQ=255)`  
                                                                                                                               `aaaaGGCTTGGGGTCCAGGCGATGGCTACACGACCATCGAGTTACGTCGAGTAACCTCCACTACCGACCGATTGGTTGACTTTACGGATGGTTCAATCCTCAGCGCGTATGACCTTAACGTCGCTCAGATTCAAATGATGCACGTAGCGGAAGAGGCCCGTGACCTCACTGCGGTTACTATCAGTGTCAATAACGATGGTCACTTGGATGCTCGTGGTCGTCGAATTGTGACCCTAGCGAACGACGTGGATGACCGCGATGCTGTTCCGTTTGGTCAACTAAAGACCATGGACCAGAACTc                                                                                                                                                                         <  1:214727/300‑1 (MQ=255)`  
                                                                                                                                   `ggCTTGTGGTCCAGCCGATGGCTACACGACCATCGAGTTACGTCGAGTAACCTCCACTACCGACCGATTGGTTGACTTTACGGATGGTTCAATCCTCCGCGCGTATGACCTTAACGTCGCTCAGATTCAAACGATGCACGTAGCGGAAGAGGCCCGTGACCTCACTGCGGATACTATCGGTGTCAATAACGATGGTCACTTGGATGCTCGTGGTCGTCGAATTGTGAACCTAGCGAACGCCGTGGATGACCGCGATGCTGTTCCGTTTGGTCAACTAAAGACCATGGACCAGAACTCATgg                                                                                                                                                                     >  1:659431/1‑301 (MQ=255)`  
                                                                                                                                   `ggCTTGGGGTCCAGCCGATGGCTACACGACCATCGAGTTACGTCGAGTAACCTCCACTACCGACCGATTGGTTGACTTTACGGATGGTTCAATCCTCCGCGCGTATGACCTTAACGTCGCTCAGATTCAAACGATGCACGTAGCGGAAGAGGCCCGTGACCTCACTGCGGATACTATCGGTGTCAATAACGATGGTCACTTGGATGCTCGTTGTCGTCGAATTGTGAACCTAGCGAACGCCGTGGATGACCGCGATGCTGTTCCGTTTGGTCAACTAAAGACCATGGACCAGAACTCATgg                                                                                                                                                                     >  1:531297/1‑301 (MQ=255)`  
                                                                                                                                         `gggTCCAGCCGATGGCTACACGACCATCGAGTTACGTCGAGTAACCTCCACTACCGACCGATTGGTTGACTTTACGGATGGTTCAATCCTCCGCGTGTATGACCTTAACGTCGCTCAGATTCAAACGATGCACGTAGCGGAAGAGGCCCGTGACCTCACTGCGGATCCTATCGGTGTCAATAACGATGGTCACTTGGATGCTCGTGGTCGTCGAATTGTGAACCTAGCGAACGCCGTGGATGACCGCGATGCTGTTCCGTTTGGTCAACTAAAGACCATGAACCAGAACTCATGGAAAgaa                                                                                                                                                               >  1:381659/1‑299 (MQ=255)`  
                                                                                                                                         `gggTCCAGCCGATGGCTACACGACCATCGAGTTACGTCGAGTAACCTCCACTACCGACCGATTGGTTGACTTTACGGATGGTTCAATCCTCCGCGCGTATGACCTTAACGTCGCTCAGATTCAAACGATGCACGTAGCGGAAGAGGCCCGTGACCTCACTGCGGATACTATCGGTGTCAATAACGATGGTCTCTTGGATGCTCGTGGTCGTCGAATTGTGAACCTAGCGAACGCCGTGGATGACCGAGATGCTGTTCCGTTTGGTCAACTAAAGACCATGGACCAGAACTCATGGCAAGCa                                                                                                                                                               <  1:415204/301‑1 (MQ=255)`  
                                                                                                                                                `gCCGATGGCTACACGACCATCGAGTTACGTCGAGTAACCTCCACTACCGACCGATTGGTTGACTTTACGGATGGTTCAATCCTCCGCGCGTATGACCTTAACGTCGCTCAGATTCAAACGATGCACGTAGCGGAAGAGGCCCGTGACCTCACTGCGGATACTATCGGTGTCAATAACGATGGTCACTTGGATGCTCGTGGTCGTCGAATTGTGAACCTAGCGAACGCCGTGGATGACCGCGATGCTGTTCCGTTTGGTCAACTAAAGACCATGGACCAGAACTCATGGCAAGCACGTAATg                                                                                                                                                        <  1:617353/301‑1 (MQ=255)`  
                                                                                                                                                     `tGGCTACACGACCATCGAGTTACGTCGAGTAACCTCCACTACCGACCGATTGGTTGACTTTACGGATGGTTCAATCCTCCGCGCGTATGACCTTAACGTCGCTCAGATTCAAACGATGCACGTAGCGGAAGAGGCCCGTGACCTCACTGCGGATACTATCGGTGTCAATAACGATGGTCACTTGGATGCTCGTGGTCGTCGAATTGTGAACCTAGCGAACGCCGTGGATGACCGCGATGCTGTTCCGTTTGGTCAACTAAAGACCATGGACCAGAACTCATGGCAAGCACGTAATGAAGcc                                                                                                                                                   <  1:37637/301‑1 (MQ=255)`  
                                                                                                                                                     `tGGCTACACGACCATCGAGTTACGTCGAGTAACCTCCACTACCGAACGATTGGTTGACTTTACGGATGGTGCAATCCTCCGCGCTTATGACCTTAACGTCGCTCAGATTCAAACGATGCACGTAGCGGAAGAGGCCCGTGACCTCACTGCGGATACTATCGGTGTCAATAACGATGGTCACTTGGATGCTCGTGGTCGTCGAATTGTGAACCTAGCGAACGCCGTGGATGACCGCGATGCTGTGCCGTTTGGTCAACTAAAGACCATTGACCAGAACTCATGGCAATCACGTAATGAAGcc                                                                                                                                                   <  1:549385/301‑1 (MQ=255)`  
                                                                                                                                                        `cTACACGACCATCGAGTTACGTCGAGTAACCTCCACTACCGACCGATTGGTTGACTTTACGGATGGTTCAATCCTCCGCGCGTATGACCTTAACGTCGCTCAGATTCAAACGATGCACGTAGCGGAAGAGGCCCGTGACCTCACTGCGGATACTATCGGTGTCAATAACGATGGTCACTTGGATGCTCGTGGTCGTCGAATTGTGAACCTAGCGAACGCCGTGGATGACCGCGATGCTGTTCCGTTTGGTCAACTAAAGACCATGGACCAGAACTCATGGCAAGCACGGAATGAAGCCTTa                                                                                                                                                >  1:212261/1‑301 (MQ=255)`  
                                                                                                                                                           `ggctcgggagatgtgtataagagacagcTCCACTACCGACCGATTGGTTGACTTTACGGATGGTTCAATCCTCCGCGCGTATGACCTTAACGTCGCTCAGATTCAAACGATGCACGTAGCGGAAGAGGCCCGTGACCTCACTGCGGATACTATCGGTGTCAATAACGATGGTCACTTGGATGCTCGTGGTCGTCGAATTGTGAACCTAGCGAACGCCGTGGATGACCGCGATGCTGTTCCGTTTGGTCAACTAAAGACCATGGACCAGAACTCATGGCAAGCACGTAATGAAGCCTTACAg                                                                                                                                             <  1:33423/274‑1 (MQ=255)`  
                                                                                                                                                           `ggctcggagatgtgtataagagacagccTCCACTACCGACCGATTGGTTGACTTTACGGATGGTTCAATCCTCCGCGCGTATGACCTTAACGTCGCTCAGATTCAAACGATGCACGTAGCGGAAGAAGCCCGTGACCTCACTGCGGATACTATCGGTGTCAATAACGATGGTCACTTGGATGCTCGTGGTCGTCGAATTGTGAACCTAGCGAACGCCGTGGATGACCGCGATGCTGTTCCGTTTGGTCAACTAAAGACCATGGACCAGAACTCATGGCAAGCACGTAATGAAGCCTTACAg                                                                                                                                             <  1:455232/275‑1 (MQ=255)`  
                                                                                                                                                              `gtgggctcggagatgtgtataagagacagcTACCGACCGATTGGTTGACTTTACGGATGGTTCAATCCTCCGCGCGTATGACCTTAACGTCGCTCAGATTCAAACGATGCACGTAGCGGAAGAGGCCCGTGACCTCACTGCGGATACTATCGGTGTCAATAACGATGGTCACTTGGATGCTCGTGGTCGTCGAATTGTGAACCTAGCGAACGCCGTGGATGACCGCGATGCTGTTCCGTTTGGTCAACTAAAGACCATGGACCAGAACTCATGGCAAGCACGTAATGAAGCCTTACAGTTc                                                                                                                                          <  1:477316/272‑1 (MQ=255)`  
                                                                                                                                                              `gACCATCGAGTTACGTCGAGTAACCTCCACTACCGACCGATTGGTTGACTTTACGGATTGTTCAATCCTCCGCGCGTATGACCTTAACGTCGCTCAGATTCAAACGATGCACGTAGAGGAAGAGGCCCATGACCTCACTGCGGATACTATCGGTGTCAATAACGATGGTCACTTGGATGCTCGTGGTCGTCGAATTGTGAACCTAGCGAACGCCGTGGATGACCGCGATGCTGTTCCGTTTGGTCAACTAAAGACCATGGACCAGAACTCATGGCAAGAACGTAATGAAGCCTTACAGTTc                                                                                                                                          <  1:761060/301‑1 (MQ=255)`  
                                                                                                                                                               `aCCATCGAGTTACGTCGAGTAACCTCCACTACCGACCGATTGGTTGACTTTACGGATGGTTCAATCCTCCGCGCGTATGACCTTAACGTCGCTCAGATTCAAACGATGCACGTAGCGGAAGAGGTCCGTGACCTCACTGCGGATACTATCGGTGTCAATAACGATGGTCACTTGGATGCTCGTGGTCGTCGAATTGTGAACCTAGCGAACGCCGTGGATGACCGCGATGCTGTTCCGTTTGGTCAACTAAAGACCATGGACCAGAACTCATGGcctgtctcttatacacatctccgagccc                                                                                                                                         >  1:682424/1‑274 (MQ=255)`  
                                                                                                                                                                  `aTCGAGTTACGTCGAGTAACCTCCACTACCGACCGATTGGTTGACTTTACGGATGGTTCAATCATCCGCGCGTATGACCTTAACGTCGCTCAGATTCAAACGATGCACGTAGCGGAAGAGGACCGTGAACTCACTGCGGATACTATCGGTGTCAATAACGATGGTCACTTGGATGCTCGTGGTCGTCGAATTGTGAACCTAGCGAACGCCGTGGATGACCGCGATGCTGTTCCGTTTGGTCAACTAAAGACCATGGACCAGAACTCATGGCAAGCACGTAAtctgtctcttatacacatct                                                                                                                                      >  1:34478/1‑282 (MQ=255)`  
                                                                                                                                                                       `gTTACGTCTAGTAACCTCCACTACCGACCGATTGGTTGACTTTACGGATGGTTAAATCCTCCACGCGTATGACCTTAACGTCGCTCAGATTCAAACGATGCACGTAGCGGAAGAGGCCCGTGACCTCACTGCGGATACTATCGGTGTCAATAACGATGGTCACTTGGATGCTCGTGGTCGTCGAATTGTGAACCAAGCGAACGCCGTGGATGACCGCGATGCTGTTCCGTTTGGTCAACTAAAGACCATGGACCAGAACTCATGGCAAGCACGTAATGAAGCCTTACAGTTCCGTaactgt                                                                                                                                 >  1:81905/1‑297 (MQ=255)`  
                                                                                                                                                                       `gTTACGTCGAGTAACCTCCACTACCGACCGATTGGTTGACTTTACGGATGGTTCAATCCTCCGCGCGTATGACCTTAACGTCGCTCAGATTCAAACGATGCACGTAGCGGAAGAGGCTCGTGACCTCACTGCGGATACTATCGGTGTCAATAACGATGGTCACTTGGATGCTCGTGGTCGTCGAATTGTGAACCTAGCGAACGCCGTGGATGACCGCGATGCTGTTCCGTTTGGTCAACTAAATACCATGGACCAGAACTCATGGCAAGCACGTAATGAAGCCTTACAGTTCCGTAATGAg                                                                                                                                 >  1:304504/1‑301 (MQ=255)`  
                                                                                                                                                                            `gTCGAGTAACCTCCACTACCGACCGATTGGTTGACTTTACGGATGGTTCAATCCTCCGCGCGTATGACCTTAACGTCGCTCAGATTCAAACGATGCACGTAGCGGAAGAGGCACGTGACCTCACTGCGGATACTATCGGTGTCAATAACGATGGTCACTTGGATGCTCGTGGTCGTCGAATTGTGAACCTAGCGAACGCTGTGGATGACCGCGATGCTGTTCCGTTTGGTCAACTAAAGACCATGGACCAGAACTCATGGCCAGCACGTAATGAAGCCTTAAAGTTCCGTAATGAGGCTgg                                                                                                                            >  1:805652/1‑300 (MQ=255)`  
                                                                                                                                                                               `gAGTAACCTCCACTACCGACCGATTGGTTGACTTTACGGATGGTTCAATCCTCCGCGCGTATGACCTTAACGTCGCTCAGATTCAAACGATGCACGTAGCGGAAGAGGCCCGTGACCTCACTGCGGATACTATCGGTGTCAATAACGATGGTCACTTGGATGCTCGTGGTCGTCGAATTGTGAACCTAGCGAACGCCGTGGATGACCGCGATGCTGTTCCGTTTGGTCAACTAAAGACCATGGACCAGAACTCATGGCAAGCACGTAATGAAGCCtgtctcttatacacatctccgagccc                                                                                                                         >  1:163324/1‑276 (MQ=255)`  
                                                                                                                                                                               `gAGTAACCTCCACTACCGACCGATTGGTTGACTTTACGGATGGTTCAATCCTCCGCGCGTATGACCTTAACGTCGCTCAGATTCAAACGATGCACGTAGCGGAAGAGGCCCGTGACCTCACTGCGGATACTATCGGTGTCAATAACGATGGTCACTTGGATGCTCGTGGTCGTCGAATTGTGAACCTAGCGAACGCCGTGGATGACCGCGATGCTGTTCCGTTTGGTCAACTAAAGACCATGGACCAGAACTCATGGCAAGCACGTAATGAAGCCTTACAGTTCCGTAATGAGGCTGAGAc                                                                                                                         <  1:521523/301‑1 (MQ=255)`  
                                                                                                                                                                                      `cTCCACTACCGACCGATTGGTTGACTTTACGGATGGTTCAATCCTCCGCGCGTATGACCTTAACGTCGCTCAGATTCAAACGATGCACGTAGCGGAAGAGGCCCGTGACCTCACTGCGGATACTATCGGTGTCAGTAACGATTGTCACTTGGATGCTCGTGGTCGTCGAATTGTGAACCTAGCGAACGCCGTGGATGACCGCGATGCTGTTCCGTTTGGTCAACTAAAGACCATGTACCAGAACTCATGGCAAGCACGTAATGAAGCCTTACAGTTCCGTAATGAGGCTGAGACTTTCAGa                                                                                                                  >  1:301299/1‑301 (MQ=255)`  
                                                                                                                                                                                        `ccACTACCGACCGATTGGTTGACTTTACGGATGGTTAAATCCTCCGCGCGTATGACCTTAACGTCGCTCAGATTCAAACGATGCACGTAGCGGAAGAGGCCCGTGACCTCACTGCGGATACTATCGGTGTCAATAACGATGGTCACTTGGATGCTCGTTGTCGTCGAATTGTGAACCTAGCGAACGCCGTGGATGACCGCGATGCTGTTCCGTTTGGTCAACTTAAGACCCTGGACCAGAACTCATGGCAAGCACGTAATGAAGCCTTACAGTTCCGTAATGAGGCTGAGACTTTCCGaac                                                                                                                >  1:180930/1‑300 (MQ=255)`  
                                                                                                                                                                                          `aCTCCCGACCGATTGGTTGACTTTACGGATGGTTCAATCCTCCGCGCGTATGACCTTAACGTCGCTCAGATTCAAACGATGCACGTAGCGGAAGAGGCCCGTGACCTCACTGCGGATACTATCGGTGTCAATAACGATAGTCACTTGGATGCTCGTGGTCGTCGACTTGTGAACCTAGCGAACGCCGTGGATGACCGCAATGCTGTTCCGTTTGGTCAACTAAAGACCATGGACCAGAACTCATGGCAAGCACGTAATGAAGCCTTACAGTTCCGTAATGAGGCTGAGACTTTCAgctgtc                                                                                                              >  1:320068/1‑296 (MQ=255)`  
                                                                                                                                                                                             `accgactgATTGGTTGACTTTACGGATGGTTCAATCCTCCGCGCGTATGACCTTAACGTCGCTCAGATTCAAACGATGCACGTAGCGGAAGAGGCCCGTGACCTCACTGCGGATACTATCGGTGTCAATAACGATGGTCACTTTGATGCTCGTGGTCGTCGAATTGTGAACCTAGCGAACGCCGTGGATGACCGCGATGCTGGTCCGTTTTGTCAACTAAAGAACATGGACCAGAACTCATGGCAAGCACGTAATGAAGCATAACAGTTCCGTAATGAGGCTGAGACTTTCAGAAACCAAg                                                                                                           >  1:554710/1‑301 (MQ=255)`  
                                                                                                                                                                                                  `ccgaTTGGTTGACTTTACGGATGGTTCAATCCTCCGCGCGTATGACCTTAACGTCGCTCAGATTCAAACGATGCACGTATCGGAAGAGGCCCGTGACCTCACTGCGGATACTATCGGTGCCAATAACGATGGTCACTTGGATGCTCGTGGTCGTCGAATTGTGAACCTAGCGAACGCCGTGGATGACCGCGATGCTGTTCCGTTTGGTCAACTAAAGACCATGGACCAGAACTCATGGCAAGCACGTAATGAAGCCTTACAGTTCCGTAATGAGGCTGAGACTTTCAGAAACCAAGCGGAg                                                                                                      >  1:175090/1‑301 (MQ=255)`  
                                                                                                                                                                                                       `tGGTTGACTTTACGGATGGTTCAATCCTCCGCGCGTATGACCTTAACGTCACTCAGATTCAAACGATGCACGTAGCGGAAGAGGCCCGTGACCTCACTGCGGATACTATCGGTGTCAATAACGATGGTCACTTGGATGCTCGTGGTCGTCGAATTGTGAACCTAGCGAACGCCGTGGATGACCGCGATGCTGTTCCGTTTGGTCAACTAAAGACCATGGACCAGAACTCATGGCAAGCACGTAATGAAGCCTTACAGTTCCGTAATGAGGCTGAGACTTTCAGAAACCAAGCGGAGGGCtt                                                                                                 <  1:689875/301‑1 (MQ=255)`  
                                                                                                                                                                                                        `ggTTGACTTTACGGATGGTTCAATCCTCCGCGCGTATGACCTTAACGTCGCTCAGATTCAAACGATGCACGTAGCGGAAGAGGCCCGTGACCTCACTGCGGATACTATCGGTGTCAATAACGATGGTCACTTGGATGCTCGTGGTCGTCGAATTGTGAACCTAGCGAACGCCGTGGATGACCGCGATGCTGTTCCGTTTGGTCAACTAAAGACCATGGACCAGAACTCATGGCAAGCACGTAATGAAGCCTTACAGTTCCGTAATGAGGCTTAGACTTTCAGAAACCAAGCGGATGGCttt                                                                                                >  1:612937/1‑301 (MQ=255)`  
                                                                                                                                                                                                            `gtataagagacaggTTCAATCCTCCGCGCGTATGACCTTAACGTCGCTCAGATTCAAACGATGCACGTAGCGGAAGAGGCCCGTGACCTCACTGCGGATACTATCGGTGTCAATAACGATGGTCACTTGGATGCTCGTGGTCGTCGAATTGTGAACCTAGCGAACGCCGTGGATGACCACGATGCTGTTCCGTTTGGTCAACTAAAGACCATGGACCAGAACTCATGGCAAGCACGTAATGAAGCCTTACAGTTCCGTAATGAGGCTGAGACTTTCAGAAACCAAGCGGAGGGCTTTAAGa                                                                                            <  1:766952/289‑1 (MQ=255)`  
                                                                                                                                                                                                             `aCTTTACGGATGGTTCAATCCTCCGCGCGTATGACCTTAACGTCGCTCAGATTCAAACGATGCACGTAGCGGAAGCGGCCCGTGACCTCACTGCGGATACTATCGGTGTCAATAACTATGGTCACTTGGATGCTCGTGGTCGTCGAATTGTGAACCTACCGCACGCCGTGGATGACCGCGATGCTGTTCCGTTTGGTCAACTAACGACCATGGACCAGAACTCATGGCAAGCACGTAATGAAGCCTTACAGTTCCGTAATGAGGCTGAGACTTTCAGAAACCAAGCGGAGGGCTTTAAGaa                                                                                           >  1:404501/1‑301 (MQ=255)`  
                                                                                                                                                                                                             `aCTTTACGGATGGTTCAATCCTCCGCGCGTATGACCTTAACGTCGCTCAGATTCAAACGATGAACGTAGCGGAAGAGGCCCGTGACCTCACTGCGGATACTATCGGTGTCAATAACGATGGTCACTTGGATGCTCGTGGTCATCGAATTGTGAACCTAGCGAACGCCGTGGATGACCGCGATGCTGTTCCGTTTGGTCAACTAAAGACAATGGACCAGAACTCATGGCAAGCACGTAATGAAGCCTTACAGTTCCGTAATGAGGCTGAGACTTTCAgctgtctcttatacacatctccgag                                                                                           >  1:499241/1‑277 (MQ=255)`  
                                                                                                                                                                                                              `cTTTACTGATGGTTCAATCCTCCGCGCGTATGACCTTAACGTCGCTCAGATTCAAACGATGCACGTAGCGGAAGAGGCCCGTGACCTCACTGCGGATACTATCGGTGTCAATAACGATGGTCACTTGGATGCTCGTGGTCGTCGAATTGTGAACCTAGCGAACGCCGTGGATGACCGCGATGCTGTTCCGTTTGGTCAACTAAAGACCATGTACCAGAACTCATGGCAAGCACGTAATGAAGCCTTACAGTTCCGTAATGAGGCTGAGACTTTCAGAAACCAAGCGGAggctgtctcttat                                                                                          >  1:291545/1‑290 (MQ=255)`  
                                                                                                                                                                                                                `ttACGGATGGTTCAATCCTCCGCGCGTATGACCTTAACGTCGCTCAGATTCAAACGATGCACGTAGCGGAAGAGGCCCGTGACCTCACTGCGGATACTATCGGTGTCAATAACGATGGTCACTTGGATGCTCGTGGTCGTCGAATTGTGAACCTAGCGAACTCCGTGGATGACCGCGATGCTGTTCCGTTTGGTCAACTAAAGACCATGGACCAGAACTCATGGCAAGCACGTAATGAAGCCTTACAGTTCCGTAATGAGGCTGAGACTTTCAgctgtctcttatacacatctccgagccc                                                                                        >  1:81553/1‑274 (MQ=255)`  
                                                                                                                                                                                                                       `ttgTTCAATCCTCCGCGCGTATGACCTTAACGTCGCTCAGATTCAAACGATGCACGTAGCGGAAGAGGCCCGTGACCTCACTGCGGATACTATCGGTGTCAATAACGATGGTCACTTGGATGCTCGTGGTCGTCGAATTGTGAACCTAGCGAACGCCGTGGATGACTGCGATGCTGTTCCGTTTGGTCAACTAAAGACCATGGACCAGAACTCATGGCAAGCACGTAATGGAGCCTTACAGTTCCGTAATGAGGCTGAGACTTTCAGAAACCAAGCGGAGGGCTTTAAGAACGAGTCCAGt                                                                                 <  1:567137/299‑1 (MQ=255)`  
                                                                                                                                                                                                                         `tgggatcggagatgtgtataagagaaagcttCGCTCAGATTCAAAAGATGCACGTAGCGGAAGAGGCCCGTGACCTCACTGCGGATACTATCGGTGTAAATAACGATGGTCCCTTGGGTGCTCGTGGTCGTCGAATTGTGAACCTAGCGATCGCCGTGGATGACCGCGATGCTGTTCCGTTTGGCCAACTAAAGACCATGGACCAGAACTCATGGCAAGCACGTAATGAAGCCTTACAGTTCCGTAATGAGGCTGAGACTTTCAGAAACCAAGCGGAGGGCTTTAAGAACGAGTCCAGTAc                                                                               <  1:635099/271‑1 (MQ=255)`  
                                                                                                                                                                                                                         `gTTCAATCCTCCGCGCGTATGACCTTAACGTCGCTCAGATTCAAACGATGCACGTAGCGGAAGAGGCCCGTGACCTCACTGCTGATATTATCGGTGTCAATAACGATGGTCACTTGGATGCTCGTGGTCGTCGAATTGTGAACCTAGCGAACGCCGTGGATGACCGCGATGCTGTTCCGTTTGGTCAACTAAAGACCATTGACCAGAACTCATGGCAAGCACGTAATGAAGGCTTACAGTTCCGTAATGAGGCTGAGACTTTCAGAAACCAAGCGGAGGGCTTTAAGAACGAGTCCAGTAc                                                                               >  1:107352/1‑301 (MQ=255)`  
                                                                                                                                                                                                                         `gTTCAATCCTCCGCGCGTATGACCTTAACGTCGCTCAGATTCAAACGATGCACGTAGCGGAAGAGGCCCGTGACCTCACTGCGGATACTATCGGTGTCAATAACGATGGTCACTTGGATGCTCGTGGTCGTCGAATTGTGAACCTAGCGAACGCCGTGGATGACCGCGATGCTGTTCCATTTGGTCAACTAAAGACCATGGACCAGAACTCATGGCAAGCACGTAATGAAGCCTTACAGTTCCGTAATGAGGCTGAGACTTTCAGAAACCAAGCGGAGGGCTTTAAGAACGAGTCCAGTAc                                                                               >  1:714604/1‑301 (MQ=255)`  
                                                                                                                                                                                                                          `ttCAATCCTCCGCGCGTATGACCTTAACGTCGCTCAGATTCAAACGATGCACGTAGCGGAAGAGGCCCGTGACCTCACTGCGGATACTATCGGTGTCAATAACGATGGTCACTTGGATGCTCGTGGTCGTCGAATTGTGAACCTAGCGAACGCCGTGGATGACCGCGATGCTGTTCCGTTTGGTCAACTAAAGACCATGGACCAGAACTCATGGCAAGCACGTAATGAAGCCTTACAGTTCCGTAATGAGGCTGAGACTTTCAGAAACCAAGCGGAGGGCTTTAAGAACGAGTCCAGTAcc                                                                              <  1:152422/301‑1 (MQ=255)`  
                                                                                                                                                                                                                               `ataagagacagTATGACCTTAACGTCGCTCAGATTCAAACGATGCACGTAGCGGAAGAGGCCCGTGACCTCACTGCGGATACTATCGGTGTCAATAACGATGGTCACTTGGATGCTCGTGGTCGTCGAATTGTGAACATAGCGAACGCCGTGGATGACCGCGATGCTGTTCCGTTTGGTCAACTAAAGACCATGGACCAGAACTCATGGCAAGCACGTAATGAAGCCTTACAGTTCCGTAATGAGGCTGAGACTTTCAGAAACCAAGCGGAGGGCTTTAAGAACGAGTCCAGTACCAACGc                                                                         <  1:631170/291‑1 (MQ=255)`  
                                                                                                                                                                                                                                        `cgTCTGACCTTAACGTCGCTCAGATTCAAACGATGCACGTAGCGGAAGAGGCCCGTGACCTCACTGCGGATACTATCGGTGTCAATAACGATGGTCACTTGGATGCTCGTGGTCGTCGAATTGTGAACCTAGCGAACGCCGTGGATGACCGCGATGCTGTTCCGTTTGGTCAACTAAATACCATGGACCAGAACTCATGGCAAGCACGTAATGAAGCCTTACAGTTCCGTAATGAGGCTGAGACTTTCAGAAACCAAGCGGAGGGCTTTAAGAACGAGTCCAGTAcctgtctcttatacac                                                                >  1:7439/1‑287 (MQ=255)`  
                                                                                                                                                                                                                                        `cgTATGACCTTAACGTCGCTCAGATTCAAACGATGCACGTAGCGGAAGAGGCCCGTGACCTCACTGCGGATACTATCGGTGTCAATAACGATGGTCACTTGGATGCTCGTGGTCGTCGAATTGTGAACCTAGCGAACGCCGTGGATGACCGCGATGCTGTTCCGTTTGGTCAACTAAAGACCATGGACCAGAACTCATGGCAAGCACGTAATGAAGCCTTACAGTTCCGTAATGAGGCTGAGACTTTCAGAAACCAAGCGGAGGGCTTTAAGAACGAGTCCAGTACCAACGCTACGAacac                                                                <  1:471467/301‑1 (MQ=255)`  
                                                                                                                                                                                                                                        `cgTATGACCTTAACGTCGCTCAGATTCAAACGATGCACGTAGCGGAAGAGGCCCGTGACCTCACTGCGGATACTATCGGTGTCAATAACGATGGTCACTTGGATGCTCGTGGTCGTCGAATTGTGAACCTAGCGAACGCCGTGGATGACCGCGATGCTGTTCAGTTTGGTCAACTAAAGACCATGGACCAGAACTCATGGCAAGCACGTAATGAAGCCTTACAGTTCCGTAATGAGGCTGAGACTTTCAGAAACCAAGCGGATGGCTTTAAGAACGAGTCCAGTACCAACGCTACGAacac                                                                >  1:754665/1‑301 (MQ=255)`  
                                                                                                                                                                                                                                        `cgTATGACCTTAACGTCGCTAAGATTCAAACGATGCACGTAGCGGAAGAGGCCCGTGACCTCACTGCGGATACTATCGGTGTCAATAACGATGGTCACTTGGATGCTCGTGGTCGTCGAATTGTGAACCTAGCGAACGCCGTGGATGACCGCGATGCTGTTCCGTTTGGTCAACTAAAGACCATGGACCAGAACTCATGGCAAGCACGTAATGAAGCCTTACAGTTCCGTAATGAGGCTGAGACTTTCAGAAACCAAGCGGAGGGCTTTAAGAACGAGTCCAGTAcctgtctcttatacac                                                                >  1:437118/1‑287 (MQ=255)`  
                                                                                                                                                                                                                                           `aTGACCTTAACGTCGCTCAGATTCAAACGATGCACGTAGCGGAAGAGGCCCGTGACCTCACTGCGGATACTATCGGTGTCAATAACGATGGTCACTTGGATGCTCGTGGTCGTCGAATTGTGAACATAGCGAACGCCGTGGATGACCGCGATGCTGTTCCGTTTGGTCAACTAAAGACCATGGACCAGAACTCATGGCAAGCACGTAATGAAGCCTTACAGTTCCGTAATGAGGCTGAGACTTTCAGAAACCAAGCGGAGGGCTTTAAGAACGAGTCCAGTACCAACGCTACGAACACaaa                                                             <  1:575889/301‑1 (MQ=255)`  
                                                                                                                                                                                                                                               `ccTTGACGTCGCTCAGATTCAAACGATGCACGTAGCGGAAGAGGCCCGTGACCTCACTGCGGATACTATCGGTGTCAATAACGATGGTCACTTGGATGCTCGTGGTCGTCGAATTGTGAACCTAGCGAACGCCGTGGATGACCGCGATGCTGTTCCGTTTGGTCAACTAAAGACCATGGACCAGAACTCATGGCAAGCACGTAATGAAGCCTTACAGTTCCGTAATGAGGCTGAGACTTTCAGAAACCAAGCGGAGGGCTTTAAGAACGAGTCCAGTACCAACGCTACGAACACAAAGCAg                                                         >  1:719924/1‑301 (MQ=255)`  
                                                                                                                                                                                                                                               `ccTTAACGTCGCTCAGATTCAAACGATGCACGTAGCGGAAGAGTCCCGTGACCTCACTGCGGATACTATCGGTGTCAATAACGATGGTCACTTGGATGCTCGTGGTCGTCGAATTGTGAACCTAGCTAACGCCGTGGATGACCGCGATGCTGTTCCGTTTGGTCAACTAAAGACCATGGACCAGAACTCATGGCAAGCACGTAATGAAGCCTTACAGTTCCGTAATGAGGCTGAGACTTTCAGAAACCAAGCGGAGGGCTTTAAGAACGAGTCCAGTACCAACGcctgtctcttatacacc                                                         >  1:592614/1‑285 (MQ=255)`  
                                                                                                                                                                                                                                               `ccTTAACGTCGCTCAGATTCAAACGATGCACGTAGCGGAAGAGGCCCGTGACCTCACTGCGGATACTATCGGTGTCAATAACGATGGTCACTTGGATGCTCGTGGTCGTCGAATTGTGAACCTAGCGAACGCCGTGGATGACCGCGATGCTGTTCCGTTTGGTCAACTAAAGACCATGGCCCAGAACTCATGGCAAGCACGTAATGAAGCCTTACAGTTCCGTAATGAGGCTGAGACTTTCAGAAACCAAGCGGAGGGCTTTAAGAACGAGTCCAGTACCAACGCTACGAACACAAAGCAg                                                         >  1:228318/1‑301 (MQ=255)`  
                                                                                                                                                                                                                                                `cTTAACGTCGCTCAGATTCAAACGATGCACGTAGCGGAAGAGGCCCGTGACCTCACTGCGGATACTATCGGTGTCAATAACGATGGTCACTTGGATGCTCGTGGTCGTCGAATTGTGAACATAGCGAACGCCGTGGATGACCGCGATGCTGTTCCGTTTGGTCAACTAAAGACCATGGACCAGAACTCATGGCAAGCACGTAATGAAGCCTTACAGTTCCGTAATGAGGCTGAGACTTTCAGAAACCAAGCGGAGGGCTTTAAGAACGAGTCCAGTACCAACGCTACGAACACAAAGCAGt                                                        <  1:390428/301‑1 (MQ=255)`  
                                                                                                                                                                                                                                                  `tAACGTCGCTCAGATTCAAACGATGCACGTAGCGGAAGAGGCCCGTGACCTCACTGCGGATACTATCGGTGTCAATAACGATGGTCACTTGGATGCTCGTGGTCGTCGACTTGTGCACCTAGCGAACGCCGTTGATGACCGCGATGCTGTTCCGTTTGGTCAACTAAAGACCATGGACCAGAACTCCTGGCAAGCACGTCATGAAGCCTTACCGTTCCGTAATGATGCTGAGACTTTCAGAAACCAAGCTGAGTGCTTTAAGAACGAGTCCAGTACCAACGCTACGCACACCAAGCCGTgg                                                      >  1:427098/1‑301 (MQ=255)`  
                                                                                                                                                                                                                                                              `gATTCAAATGATGCACGTAGCGGAAGAGGCCCGTGACCTCACTGCGGATACTATCGGTGTCAATAACGATGGTCACTTGGATGCTCGTGGTCGTCGAATTGTGAACCTAGCGAACGCCGTGGATGACCGCGATGCTGTTCCGTTTGGTCAACTAAAGACCATGGACCAGAACTCATGGCAAGCACGTAATGAAGCCTTACAGTTCCGTAATGAGGCTGAGACTTTAAGAAACCAAGCGGAGGGCTTTAAGAACGAGTCCAGTACCAACGCTACGAAAACAAAGCAGTGGCGCGATGAGAcc                                          >  1:373130/1‑301 (MQ=255)`  
                                                                                                                                                                                                                                                              `gATTCAAACGATGCACGTAGCGGAAGAGGCCCGTGACCTCACTGCGGATACTATCGGTGTCAATAACGATGGTCACTTGGATGCTCGTGGTCGTCGAATTGTGAACCTAGCGAACGCCGTGGATGACCGCGATGCTGTTCCGTTTGGTCAACTAAAGACCATGGACCAGAACTCATGGCAAGCACTTAATGAAGCCTTACAGTTCCGTAATGAGGCTGAGACTTTCAGAAACCAAGCGGATGGCTTTAAGAACGAGTCCAGTACCACCGCTACGAACACAAAGAAGTGGCGCGATgagaac                                          >  1:480274/1‑299 (MQ=255)`  
                                                                                                                                                                                                                                                              `gATTCAAACGATGCACGTAGCGGAAGAGGCCCGTGACCTCACTGCGGATACTATCGGTGTCAATAACGATGGTCACTTGGATGCTCGTGGTCGTCGAATTATGAACCTAGCGAACGCCGTGGATGACCGCGATGCTGTTCCGTTTGGTCAACTAAAGACCATGGACCAGAACTCATGGCAAGCACGTAATGAAGCCTTACAGTTCCGTAATGAGGCTGAGACTTTCAGAAACCAAGCGGAGGGCTTTAAGAACGAGTCCAGTACCAACGCTACGAACACAAAGCAGTCGCGCGATGAGAcc                                          >  1:356868/1‑301 (MQ=255)`  
                                                                                                                                                                                                                                                                   `aaaCGATGCACGTAGCGGAAGAGGCCCGTGACCTCACTGCGGATACTATCGGTGTCAATAACGATGGTCACTTGGATGCTCGTGGTCGTCGAATTGTGAACCTAGCGAACGCCGTGGATGACCGCGATGCTGTTCCGTTTGGTCAACTAAAGACCATGGACCAGAACTCATGGCAAGCACGTAATGAAGCCTTACAGTTCCGTAATGAGGCTGAGACTTTCAGAAACCAAGCGGAGGGCCTTAAGAACTAGTCCAGTACCAACGCTACGAACACAAAGCAGTGGCGCGATGAGACCAAggg                                     <  1:430196/301‑1 (MQ=255)`  
                                                                                                                                                                                                                                                                                 `gCGGAAGAGGCCCGTGACCTCACTGCGGATACTATCGGTGTCAATAACGATGGTCACTTGGATGCTCGTGGTCGTCGAATTGTGAACCTAGCGAACGCCGTGGATGACCGCGATGCTGTTCCGTTTGGTCAACTAAAGACCATGGACCAGAACTCATGGCAAGCACGTAATGAAGCATTACAGTTCCGTAATGAGGCTGAGACTTTCAGAAACCAAGCGGAGGGCTTTAAGAACGAGTCCAGTACCAACGCTACGAACACAAAGCAGTGGCGCGATGAGCCCAAGGGTTTCCGAGACGAAg                       >  1:649145/1‑301 (MQ=255)`  
                                                                                                                                                                                                                                                                                  `cGGAAGAGGCCCGTGACCTCACTGCAGATACTATCGGTGTCAATAACGATGGTCACTTGGATGCTCGTGGTCGTCGAATTGTGAACCTAGCGAACGCCGTGGATGACCGCGATGCTGTTCCGTTTGGTCAACTAAAGACCATGGACCAGAACTCATGGCAAGCACGTAATGAAGCCTTACAGTTCCGTAATGAGGCTGAGACTTTCAGAAACCAAGCGGAGGGCTTTAAGAACGAGTCCAGTACCAACGCTACGAACACAAAGCAGTGGCGCGATGAGACCAAGGGTTTCCGAGACGAAGc                      <  1:92399/301‑1 (MQ=255)`  
                                                                                                                                                                                                                                                                                           `cccGTGACCTCACTGCGGATACTATCGGTGTCAATAACGATGGTCACTTGGATGCTCGTGGTCGTCGAATTGTGAACCTAGCGAACGCCGTGGATGACCGCGATGCTGTTCCGTTTGGTCAACTAAAGACCATGGACCAGAACTCATGGCAAGCACGTAATGAAGCCTTACAGTTCCGTAATGAGGCTGAGACTTTCAGAAACCAAGCGGAGGGCTTTAAGAACGAGTCCAGTACCAACGCTACGAACACAAAGCAGTGGCGCGATGAGACCAAGGGTTTCCGAGACGAAGCCAAGCGGtt             >  1:460078/1‑301 (MQ=255)`  
                                                                                                                                                                                                                                                                                                 `aCCTCACTGCGGATACTATCGGTGTCAATAACGATGGTCACTTGGATGCTCGTGGTCGTCGAATTGTGAACCTAGCGAACGCCGTGGATGACCGCGATGCTGTTCCGTTTGGTCAACTAAAGACCATGGACCAGAACTCATGGCAAGCACGTAATGAAGCCTTACAGTTCCGTAATGAGGCTGAGACTTTCAGAAACCAAGCGGAGGGCTTTAAGAACGAGTCCAGTACCAACGCTACGAACACAAAGCAGTGGCGCGATGAGACCAAGGGTTTCCGAGACGAAGCCAAGCGGTTCAAgga       >  1:780967/1‑299 (MQ=255)`  
                                                                                                                                                                                                                                                                                                 `aCCTCACTGCGGATACTATCGGTGTCAATAACGATGGTCACTTGGATGCTCGTGGTCGTCGAATTGTGAACCTAGCGAACGCCGTGGATGACCGCGATGCTGTTCCGTTTGGTCAACTAAAGACCATGGACCAGAACTCATGGCAAGCACGTAATGAAGCCTTACAGTTCCGTAATGAGGCTGAGACTTTCAGAAACCAAGCGGAGGGCTTTAAGAAAGAGTCCAGTACCAACGCTACGAACACAAAGCAGTGGCGCGATGAGACCAAGGGTTTCCGAGACGAAGCCGAGCGGTTCAAGaa       >  1:112128/1‑301 (MQ=255)`  

                                                                                                                                                                                                                                                                                                         `|`                                                                                                                                                                                                                                                                                                           
`GATTTTAATATCCCGTTTGAGTATCTAGCCCGTAAGTTCGTAGTGGTAACTCTTATTGGTGTAGACCGAAAGGTCCTTACGATTAATACAGACTATCGCTTTGCTACACGTACTACTATCTCTCTGACAAAGGCTTGGGGTCCAGCCGATGGCTACACGACCATCGAGTTACGTCGAGTAACCTCCACTACCGACCGATTGGTTGACTTTACGGATGGTTCAATCCTCCGCGCGTATGACCTTAACGTCGCTCAGATTCAAACGATGCACGTAGCGGAAGAGGCCCGTGACCTCACTACGGATACTATCGGTGTCAATAACGATGGTCACTTGGATGCTCGTGGTCGTCGAATTGTGAACCTAGCGAACGCCGTGGATGACCGCGATGCTGTTCCGTTTGGTCAACTAAAGACCATGAACCAGAACTCATGGCAAGCACGTAATGAAGCCTTACAGTTCCGTAATGAGGCTGAGACTTTCAGAAACCAAGCGGAGGGCTTTAAGAACGAGTCCAGTACCAACGCTACGAACACAAAGCAGTGGCGCGATGAGACCAAGGGTTTCCGAGACGAAGCCAAGCGGTTCAAGAATACGG  >  NC_001604.1/34678‑35272`

  

Alignment Legend

Aligned base mismatch/match (shaded by quality score): `ATCG/ATCG` `< 3 ≤` `ATCG/ATCG` `< 12 ≤` `ATCG/ATCG` `< 25 ≤` `ATCG/ATCG` `< 36 ≤` `ATCG/ATCG` `< 38 ≤` `ATCG/ATCG`

Unaligned base: `atcg`    Masked matching base: `atcg`    Alignment gap: `‑`    Deleted base: `‑`
